# Low Vitamin D Level in Saudi Women With Polycystic Ovary Syndrome

**DOI:** 10.3389/fnut.2021.611351

**Published:** 2021-04-12

**Authors:** Iman Abdullah Bindayel

**Affiliations:** Department of Community Health Sciences, College of Applied Medical Sciences, King Saud University, Riyadh, Saudi Arabia

**Keywords:** BMI, females, polycystic ovary syndrome, vitamin D, 25-dihydroxyvitamin D

## Abstract

Polycystic ovary syndrome (PCOS) is a common endocrine disorder in women of reproductive age. In this study, serum 25-hydroxyvitamin D levels were compared between women with and without PCOS and with regard to anthropometric indices and lipid and glucose biomarkers. Thirty-one women with PCOS and 75 controls answered a questionnaire on vitamin D, in addition to general health and lifestyle. The patients with PCOS had lower vitamin D levels (*p* < 0.05), a significantly higher rate of obesity (*p* < 0.05), and significantly higher serum triglyceride levels than did controls. The number of patients with PCOS consumed milk and dairy products (*p* < 0.05) and exposed to sun (*p* < 0.006) were lower compared to controls. Triglyceride levels were significantly correlated with body mass index (BMI); vitamin D level was not significantly correlated with anthropometrical or biochemical variables. These results affirm that vitamin D levels are lower in women with PCOS; however, despite the significantly higher proportion of obesity among patients with PCOS, hypovitaminosis was not associated with BMI. The relationship between body composition and vitamin D in PCOS and the effect of vitamin D correction on metabolic and hormonal parameters associated with PCOS must be assessed in future trials.

## Introduction

Polycystic ovary syndrome (PCOS) is an endocrine disorder that affects women of reproductive age. It is characterized by irregular menstruation, ovarian dysfunction, hyperandrogenism, and multiple ovarian cysts (1). The worldwide prevalence of PCOS among women of reproductive age is ~6–12% (2). The prevalence of PCOS in Saudi Arabia is currently unknown.

Polycystic ovary syndrome is characterized by a pattern of hormonal imbalances and metabolic dysregulation, including compensatory hyperinsulinemia, insulin resistance, and dyslipidemia, all of which contribute to the pathogenesis (3). In addition, adiposity is highly prevalent among women with PCOS and is often associated with insulin resistance (4). Women with PCOS are pre-disposed to long-term health diseases, such as type 2 diabetes, central obesity, hyperinsulinemia, cardiovascular disease, anovulation, and endometrial cancer (5).

A growing body of evidence has linked higher prevalence of PCOS to vitamin D deficiency (6, 7). Vitamin D is a steroid hormone that is endogenously synthesized through skin exposure to solar ultraviolet light; however, <10–20% is derived from diet (5). The active form 1,25-dihydroxyvitamin D acts on its respective receptor (vitamin D receptor), which is present at multiple locations throughout the body (intestine, breast, bones, pancreas, kidney, and immune cells), to modulate the organ metabolism and function. In addition, vitamin D upregulates insulin synthesis and secretion by pancreatic cells (5). In women affected by PCOS, a suboptimal vitamin D level (<20 ng/mL) was found to be linked with several risk factors associated with PCOS, including hyperglycemia; increased scores on the homeostatic model assessment for insulin resistance (HOMA-IR); increases in levels of total cholesterol, low-density lipoprotein cholesterol (LDL-C), triglyceride (TG), and fasting plasma glucose levels; and a decrease in high-density lipoprotein cholesterol (HDL-C) level (8–12). Saudi women are at higher risk for developing vitamin D deficiency (13, 14). According to a national study, ~50% of students and 44% of employees in Saudi schools have vitamin D deficiency in the Central, Eastern, and Western regions and that the risk was higher for girls and women (14).

The potential metabolic dysregulation suspected to be caused by vitamin D deficiency in PCOS has recently been the subject of intense research. Several cross-sectional and case–control studies demonstrated lower vitamin D levels in patients with PCOS cases than in controls (6, 8, 12, 15–18); however, other studies have demonstrated no differences (11, 19–21). Such discrepancies are related to variation in study design, including sample size and methodological choice, or are related to the heterogeneity in case characteristics, such as patients' age, body mass index (BMI), skin color, diet, and physical activity. Indeed, (9) suggested a relationship between obesity and decreased levels of 25-hydroxyvitamin D. The effect of obesity on vitamin D–binding protein levels has been established and may partly account for the suboptimal level of serum vitamin D in obese patients with PCOS. Therefore, BMI should be considered in order to allow more accurate assessment of the association between vitamin D and PCOS.

In addition, obesity, particularly abdominal obesity, was found to negatively affect insulin sensitivity in patients with PCOS (18). This existing link may be directly attributable to obesity, rather than to the presence of PCOS. However, insufficient vitamin D may contribute to the worsening of insulin resistance in patients with PCOS (16). A recent meta-analysis of randomized control trials revealed that short-term vitamin D supplementation was associated with improvement in insulin resistance, total cholesterol level, and LDL-C level (22).

Overall, the association between vitamin D and PCOS risk factors and symptoms is an active area of growing research. Data related to vitamin D and PCOS in Saudi Arabia have been generated by a few studies in the western region of the kingdom. Well-designed studies are needed to measure vitamin D level and clarify its relation to PCOS-related metabolic impairments, including obesity and lipid and glucose indices in PCOS. Therefore, the primary objective of this study was to measure vitamin D concentrations in Saudi women with PCOS who visited the gynecology outpatient clinics at a tertiary hospital and to compare those concentrations with those of women who did not have PCOS (controls). In addition, vitamin D was assessed in relation to anthropometric measures such as waist-to-hip ratio (WHR), BMI, and metabolic indices (glucose and lipid profiles) in participants with PCOS and controls. The hypotheses were that vitamin D levels would be lower in participants with PCOS than in controls and that depletion in vitamin D level would be associated with adiposity and metabolic impairment in women with PCOS who were living in Riyadh, Saudi Arabia.

## Materials and Methods

This cross–sectional study was conducted with 104 women aged 15–55 years who did or did not have PCOS. They were identified primarily in an outpatient gynecology department at a tertiary hospital in Riyadh, Saudi Arabia, between February and May 2018. The protocol was approved by the Institutional Review Board (IRB) of King Saud University Medical City. A total of 31 women with PCOS (the PCOS group) and 73 controls (the control group) read and signed a consent form. Selection of participants was based on the three Rotterdam criteria, two of which confirm the diagnosis: oligo-ovulation, anovulation, or both; clinical or biochemical signs of hyperandrogenism; and polycystic ovaries as determined on ultrasonography (23). The exclusion criteria were pregnancy, hypothyroidism, renal diseases, Cushing's syndrome, congenital adrenal hyperplasia, hyperprolactinemia, androgen-secreting tumors, severe comorbidities, intake of pills that alter carbohydrate or lipid metabolism; all patients were screened for these criteria during examination. The control group consisted of 73 healthy and regularly menstruating women without clinically apparent hyperandrogenism. All women self-completed a vitamin D related dietary and lifestyle habits questionnaire. The questionnaire is designed to quantify the number of women using supplement, sunscreen, exposing to sun, performing physical activity, and consuming vitamin D rich sources.

### Anthropometrics

The following anthropometric data were collected from each participant: weight, height, waist circumference, and hip circumference. Weight (in kilograms) was measured with Tanita BWB-800 electronic scale (Arlington Heights, Illinois, USA). Participants were weighed twice wearing light clothing and no shoes and the two measurements were averaged. Height was collected using a stadiometer (Seca 213 Portable Measuring Rod, Seca Corporation, Hanover, MD). Participants were measured barefoot on a flat surface and heights were recorded in centimeters to the nearest tenth. The waist circumference was measured using a measuring tape while participants were standing, midway between the lower costal margin and the iliac crest. Hip circumference was measured while participants were standing, at the maximum circumference over the buttocks. To calculate waist to hip circumference (WHR), the waist circumference was divided by the hip circumference. BMI was calculated as the weight (in kilograms) divided by the height squared (in meters). A normal BMI-value is between 18 and 24.99 kg/m^2^; overweight is between 25 and 29.9 kg/m^2^; and obesity is a BMI ≥ 30 kg/m^2^.

### Biochemical Analysis

Blood samples were collected in the morning after participants fasted overnight, during the early follicular phase of each patient's spontaneous or progestin-induced menstrual cycle. Blood samples were sent to the Hematology and Biochemical Department at the hospital for measurement of serum 25-hydroxyvitamin D, in addition to hormonal [thyroid function test: thyroid-stimulating hormone (TSH), free thyroxine] and metabolic variables [fasting plasma glucose, glycolated hemoglobin (HbA1c), total cholesterol, HDL-C, LDL-C, and TG]. Total serum 25-hydroxyvitamin D analysis was performed with a Cobas e 411 automated analyzer (Roche Diagnostics, Indianapolis, IN, USA). Vitamin D deficiency was defined as a serum level of 25-hydroxyvitamin D level of <30 nmol/L; insufficiency, as a serum level between 30 and 50 nmol/L; and sufficiency, as a serum level higher than 50 nmol/L (24).

### Statistical Analysis

SPSS version 22.0. software (SPSS Inc., Chicago, IL, USA) was used to perform statistical analysis. Continuous data were calculated as means ± standard deviations. Nominal variables were expressed as numbers and percentages. The chi-square-test was used to compare the controls and the patients with PCOS with regard to all nominal variables. Student's *t*-test for two independent samples was used to compare the two groups with regard to all continuous variables (after confirmation of normal distribution). Difference in vitamin D level according to BMI subcategories in the two groups was tested first with analysis of variance and then a *post-hoc t*-test. Pearson's correlation coefficient (*r*) was used to test the correlation between continuous variables.

## Results

Data from 31 women with PCOS and 73 controls were included in the analyses. Anthropometric and metabolic parameters are listed in Table 1. Each woman was further categorized as normal, overweight, or obese according to her BMI. The BMI distribution among the women with PCOS differed significantly from that among the controls (*p* < 0.05; Table 1), whereby the percentage of the PCOS group who were obese (58%) was higher than that among the controls (33%).

**Table 1:** Clinical and biochemical features of women with PCOS and controls.

**Parameter**	**Control**	**PCOS**	***p-*value**
Number	*n* = 73 (70%)	*n* = 31 (30%)	
Age (years)	26 ± 4.3	27 ± 5.3	NS
BMI (kg/m^2^)	28.0 ± 6.3	29.4 ± 5.4	NS
Normal (BMI, 18–24.99)	*n* = 18 (25%)	*n* = 6 (20%)	0.049^*^
Overweight (BMI, 25–29.99)	*n* = 31 (42%)	*n* = 7 (23%)	
Obese (BMI ≥30)	*n* = 24 (33%)	*n* = 18 (58%)	
WHR	0.798 ± 0.14	0.829 ± 0.08	NS
25-hydroxyvitamin D (nmol/L)	61.01 ± 33.9	49.20 ± 22.5	0.041
Sufficient	*n* = 39 (53%)	*n* = 14 (45%)	NS^*^
Insufficient	*n* = 21 (28%)	*n* = 11 (36%)	
Deficient	*n* = 14 (19%)	*n* = 6 (19%)	
Fasting plasma glucose (mmol/L)	5.75 ± 1.1	6.2 ± 2.3	NS
HbA1c (mmol/L)	5.6 ± 1.1	6.15 ± 2.31	NS
Free thyroxine (mmol/L)	14.7 ± 4.0	15.7 ± 2.5	NS
TSH (mmol/L)	4.53 ± 6.6	3.6 ± 3.9	NS
Total cholesterol (mmol/L)	4.88 ± 1.71	4.73 ± 1.14	NS
HDL-C (mmol/L)	1.75 ± 1.21	1.68 ± 0.61	NS
LDL-C (mmol/L)	2.70 ± 0.92	2.52 ± 0.82	NS
TG (mmol/L)	1.06 ± 0.5	1.83 ± 1.46	0.044

*Results are shown as means ± standard deviations for continuous variables. Values are significant at p < 0.05*;

* *p < 0.05 with the chi-square-test. BMI, body mass index; HbA1c, glycolated hemoglobin; HDL-C, high-density lipoprotein-cholesterol; LDL-C, low-density lipoprotein-cholesterol; NS, not significant; PCOS, polycystic ovary syndrome; TG, triglyceride; TSH, thyroid-stimulating hormone; WHR, waist-to-hip ratio*.

### Vitamin D Level

The serum 25-hydroxyvitamin D levels were significantly lower in the PCOS group than in the control group (*p* < 0.05, Table 1), and serum triglyceride levels were higher in the PCOS group than in the control group (*p* < 0.05). There were no differences in the other measured parameters (Table 1).

The women's vitamin D levels were categorized as sufficient, insufficient, or deficient (Table 1). The percentages of women with sufficient, insufficient, or deficient levels did not differ between the PCOS and control groups. Vitamin D concentration was not significantly correlated with any of the measured variables.

### Body Mass Index

Grouping of participants according to BMI revealed no difference in vitamin D levels among the BMI groups in the PCOS group and among those in the control group (Table 2). BMI was positively correlated with TG level (*R* = 0.272; *p* < 0.05). In addition, grouping of participants according to vitamin D levels (sufficient, insufficient, and deficient) revealed no differences in the ratio of lean and obese participants in the PCOS and control groups (data not shown).

**Table 2:** Vitamin D level according to body mass index subcategory in patients with PCOS and controls.

**Body mass index**	**Control**	**PCOS**
Normal (BMI, 18–24.99)	57.97 ± 32	39.01 ± 18.86
Overweight (BMI, 25–29.99)	65.93 ± 37.39	55.06 ± 24.03
Obese (BMI ≥30)	59.22 ± 31.02	50.28 ± 23.09

*Results are shown as means ± standard deviations. No significant difference according to analysis of variance (p > 0.05). PCOS, polycystic ovary syndrome*.

### Vitamin D Questionnaire Output

The PCOS and control groups were assessed on the basis of their responses to questions related to vitamin D (Table 3). The ratio of participants who reported no exposure to sun was significantly lower in the PCOS group than in the control group (*p* < 0.0001). In addition, only one third of the participants in the PCOS group reported daily intake of milk products, in comparison with half of the control group. There was no difference between the groups in physical activity, use of sunscreen, use of vitamin D supplements, or knowledge about symptoms and sources of vitamin D.

**Table 3:** Responses to lifestyle and dietary questionnaire by women with PCOS and controls.

**Participants**	***N***	**Yes (%)**	**No (%)**	**Sometimes (%)**	**Chi-square *p*-value**
**Sun exposure**					0.0001
Controls	75	45	32	23	
With PCOS	31	6	26	68	
**Milk and dairy products daily consumption**					0.009
Controls	75	50	34	16	
With PCOS	31	29	26	45	
**Weekly fish intake**					NS
Controls	75	77	15	8	
With PCOS	31	58	26	16	
**Physical activity**					NS
Controls	75	64	9	27	
With PCOS	31	61	13	26	
**Family history of diseases**					NS
Controls	75	24	12	64	
With PCOS	31	29	3	68	
**Knowledge of symptoms of vitamin D deficiency**					NS
Controls	75	7	13	80	
With PCOS	31	16	3	81	
**Knowledge that the sun is the main source of vitamin D**					NS
Controls	75	8	27	65	
With PCOS	31	10	16	74	
**Awareness of relationship between skin color and vitamin D**					NS
Controls	75	20	36	44	
With PCOS	31	23	32	45	
**Knowledge that vitamin D varies according to sun exposure**					NS
**duration**					
Controls	75	17	12	71	
With PCOS	31	19	26	55	
**Use of vitamin D supplements**					NS
Controls	75	17	21	61	
With PCOS	31	16	26	58	
**Use of sunscreen**					NS
Controls	75	28	29	43	
With PCOS	31	39	13	48	

*Results are shown as numbers. Significance was calculated with the chi-square-test, significant at (p < 0.05). NS, not significant; PCOS, polycystic ovary syndrome*.

## Discussion

The evidence linking vitamin D to PCOS from observational studies and meta-analyses of randomized controlled studies is inconsistent (5, 22). In addition, studies conducted in Saudi Arabia, particularly in the Central region (17, 25), have been limited. This study is among the first in which vitamin D levels have been assessed in patients with PCOS in Riyadh, the capital city of the kingdom. The results affirm that the vitamin D level was significantly lower in women with PCOS than in those without. This finding is in agreement with those of studies conducted in different countries (8, 9, 15, 18, 26, 27). It is not consistent with that of a study of women with PCOS living in Makkah, located in the Western region, in which the vitamin D levels were similar among patients and controls (25); nonetheless, a larger study in the same region demonstrated lower levels of vitamin D in women with PCOS (17). Of interest is one report in which levels of vitamin D were higher in women with PCOS than in controls; however, the sample size was very small (16 cases and 17 controls), and approximately all participants had a vitamin D level higher than 30 ng/mL [75 nmol/L; (28)].

In this study, the percentage of women with PCOS who reported exposure to sunlight was significantly lower than that of controls. A large amount of vitamin D is synthesized in the skin through exposure to sunlight. In addition, obesity is highly prevalent among women affected by PCOS (6, 8, 9), and this was the case in our study. Hypovitaminosis D in obesity was related to either accelerated storage of 25-hydroxyvitamin D in adipose tissue or a reduction in exposure to sunlight (29, 30), which resulted in insufficient vitamin D biosynthesis generated through the skin. Indeed, a study in the United States revealed a link between lower vitamin D levels and higher body weight, lower milk intake, and higher use of sunscreen (31). In a study of teenage girls in Saudi Arabia, lower vitamin D level was linked to low dairy intake and low exposure to the sun (32). In that study, the percentage of girls with PCOS who reported daily intake of dairy products was lower than that of controls. Dairy products are fortified with vitamin D; hence, inadequate intake would have contributed in part to the observed lower level of vitamin D in the girls with PCOS. Other diet and lifestyle factors such as physical activity, use of sunscreen, and knowledge about symptoms of vitamin D deficiency and sources of vitamin D did not differ between the groups. Of note, the participants in our study showed poor knowledge about the solar source of vitamin D, the effect of duration of sunlight exposure and skin tone on vitamin D synthesis, and the symptoms of vitamin D deficiency. For example, only 8% of the controls and 10% of patients knew that the sun is the main source of vitamin D in the skin. In a recently study from Jeddah, Saudi Arabia, 39.3% of participants had such knowledge; this highlights the fact that general knowledge about vitamin D deficiency is inadequate (33). This finding also indicates the need for assessments of suitable educational strategies to improve public knowledge about vitamin D and its sources.

Several researchers have investigated the association between adiposity (assessed through BMI) and vitamin D levels in women with PCOS. BMI, although not very reliable, is nonetheless universally used to indicate adiposity level. Several researchers have reported an inverse association between BMI and vitamin D levels (6, 8, 9). In addition, one study demonstrated an inverse association between BMI and WHR with serum vitamin D levels (15). However, our results demonstrated no correlation between vitamin D and either BMI or WHR in the women with PCOS; this may be related to the large heterogeneity with regard to vitamin D levels in relation to small sample size, the use of BMI to indicate adiposity, the small number (6) of women with vitamin D deficiency in the PCOS group, and methodological differences in measuring vitamin D. In line with these findings, in a few studies in which BMI was measured, no association with vitamin D deficiency in young patients with PCOS was reported (34, 35). A study that used dual-energy X-ray absorptiometry (DEXA) to measure fat mass, abdominal fat mass, and fat-free mass in patients with PCOS and measured the correlation of BMI with vitamin D; reported that although their PCOS and control groups had similar adiposity levels, vitamin D levels were lower in the PCOS group (36). Another study demonstrated that PCOS was independently associated with lower vitamin D level, regardless of other pre-disposing risk factors (37). Kensara, controlling for age and BMI, found a lower vitamin D level in lean women with untreated PCOS than in controls (17). Therefore, according to our findings and the evidence reviewed, it is possible that the reduction in vitamin D in PCOS is not affected directly by BMI; however, BMI may contribute to the spectrum of metabolic disturbances associated with the illness. For example, our finding that BMI was positively correlated with TG level, which is a common metabolic risk factor in PCOS pathogenesis and is linked to greater cardiovascular risk.

In women affected by PCOS, vitamin D depletion has been linked to higher HOMA-IR score; higher levels of glucose, total cholesterol, LDL-C, and TG; and lower HDL-C level (8–10). In this study, levels of vitamin D were low in women with PCOS; however, no association between vitamin D and lipid, glucose, or anthropometric indices were found. Results of another study indicated a downregulation in vitamin D transportation *via* its binding protein in PCOS (38), Therefore, in future studies, vitamin D transporter level should be measured in patients with PCOS.

The hypovitaminosis observed in this study should be interpreted with caution because it could be explained by the fact that the incidence of vitamin D deficiency depends on PCOS severity. The prevalence of vitamin D deficiency should be assessed with regard to PCOS development stage (severity) because it may contribute to the significant difference in vitamin D status between patients with PCOS and those without (39).

The limitations of this study include its small sample size, its observational design, dependency on patient self -reporting, and low generalizability, inasmuch as outpatients attending a tertiary hospital in Riyadh were the only participants recruited. Confounding factors such as Islamic clothing and area of living were not addressed. Regarding body composition, the study used BMI and WHC to assess adiposity, however, the use of bioelectrical impedance analysis for body composition may have been more suited. However, the study has several strengths, such as the accounting for BMI distribution, the assessment of the participants' exposure to sun, use of sunscreen, physical activity, and other vitamin D–related dietary and lifestyle habits, which may help in the interpretation of vitamin D outcome.

Overall, vitamin D deficiency is usually clinically unrecognizable, although it is implicated in the origins of several health disorders, including PCOS. It is recommended that vitamin D level be assessed in patients with PCOS. In addition, a multicenter national study that includes different regions of Saudi Arabia with a large sample is needed to confirm the finding. Moreover, prospective interventional trials may help clarify the role and causality of vitamin D in the pathogenesis of PCOS and thereby improve the diagnostic and treatment strategies for Saudi women with PCOS.

## Conclusion

In conclusion, the results of this study affirm that vitamin D levels are lower in Saudi women with PCOS than in women who do not have PCOS. This difference was not linked to BMI or other measured variables. Well-designed studies that address the reasons for such difference are warranted. All patients with PCOS must be screened for 25-hydroxyvitamin D deficiency, and appropriate replacement therapy must be instituted to prevent adverse consequences.

## Data Availability Statement

The raw data supporting the conclusions of this article will be made available by the authors, without undue reservation.

## Ethics Statement

The studies involving human participants were reviewed and approved by The Protocol was approved by the Institutional Review Board (IRB) of King Saud University Medical City. Written informed consent to participate in this study was provided by the participants.

## Author Contributions

IB: conceptualization, methodology, data analysis, resources, original draft, and final draft preparation.

## Conflict of Interest

The author declares that the research was conducted in the absence of any commercial or financial relationships that could be construed as a potential conflict of interest. The funders had no role in the design of the study; in the collection, analyses, or interpretation of data; in the writing of the manuscript, or in the decision to publish the results.
